# Self-reported risk of obstructive sleep apnea syndrome, and awareness about it in the community of 4 insular complexes comprising 41 Greek Islands

**DOI:** 10.5935/1984-0063.20220009

**Published:** 2022

**Authors:** Anastasia Adamou, Athanasios-Stefanos Giannopoulos, Christina Arvaniti, Ioannis Belios, Dimitra Dalampira, Georgios Eleftheriadis, Thomai Zinoviou, Petros Kassas, George D. Vavougios, Chrissi Hatzoglou, Konstantinos I. Gourgoulianis, Sotirios G. Zarogiannis

**Affiliations:** 1 Faculty of Medicine, University of Thessaly, Department of Physiology - Larissa - Thessaly - Greece.; 2 Faculty of Medicine, University of Thessaly, Department of Respiratory Medicine - Larissa - Thessaly - Greece.

**Keywords:** Cross-Sectional Studies, Sleep Apnea, Obstructive Sleep Apnea Syndrome, Risk Assessment

## Abstract

Obstructive Sleep Apnea Syndrome (OSAS) is a chronic disease that significantly increases morbidity and mortality of the affected population. There is lack of data concerning the OSAS prevalence in the insular part of Greece. The purpose of this study was to investigate the self-reported prevalence of OSAS in 4 Greek insular complexes comprising 41 islands, and to assess the awareness of the population regarding OSAS and its diagnosis. Our study comprised 700 participants from 41 islands of the Ionian, Cyclades, Dodecanese and Northeast Aegean island complexes that were studied by means of questionnaires via a telephone randomized survey (responsiveness rate of 25.74%). Participants were assessed by the Berlin Questionnaire (BQ) for evaluation of OSA risk, by the Epworth Sleepiness Scale (ESS) for evaluation of excessive daytime sleepiness, and by 3 questions regarding the knowledge and diagnosis of OSAS. The percentage of participants at high risk according to BQ was 27.29% and the percentage of people who were at high risk according to ESS was 15.43%. A percentage of 6.29% of the population was at high risk for OSAS (high risk both in BQ and ESS). A high percentage of 73.43%, were aware of OSAS as a syndrome however a significantly less percentage (28.00%) was aware of how a diagnosis of OSAS is established. The community prevalence of OSAS in Greek islands in combination with the low-level awareness of the OSAS diagnostic methods highlights the need for development of health promotion programs aiming at increasing the detection of patients at risk while increasing the awareness of OSAS.

## INTRODUCTION

The obstructive sleep apnea syndrome (OSAS) is a common chronic heterogeneous disorder, in which the patients experience frequent apneas or hypopneas during sleep. This is due to the repetitive collapse of the upper airway and/ or intermittent complete obstruction that interrupts normal ventilation during sleep^1^. The affected patients suffer from daytime somnolence, impaired concentration, exhaustion and a reduced quality of life^2^.

Even though the risk assessment for OSAS is based on the medical record and clinical examination, final diagnosis requires validation by polysomnography (PSG) in a sleep medicine clinic. Home sleep apnea tests can also be performed for certain patients but are generally considered less accurate than the PSG test^3^. Oximetry combined with a questionnaire has also been used as a method for exclusion from a sleep medicine clinic based diagnosis of OSAS, because of its high sensitivity^4^. Questionnaires like the Berlin questionnaire (BQ) and the Epworth Sleepiness Scale (ESS) are increasingly used to define the population at high risk for OSAS. These questionnaires have been translated and validated in several languages, including Greek, by studies conducted in sleep clinics^5,6^.

OSAS is considered a major public health issue due to its impact on patient’s health and quality of life along with comorbidities such as metabolic syndrome and cardiovascular diseases^7,8^. First-line therapy with continuous positive airway pressure (CPAP) was found to be clinically more effective than no treatment at all in both males and females, and it constitutes a relief to the financial burden associated with untreated OSAS^9^. Therefore, it is important to gather evidence regarding the prevalence of the disease in the community. There are some studies that concern several parts of the Greek population such as children and adolescents^10^, hospitalized patients^11^, railway drivers^12^ and nursing staff^13^. However, there is scarcity of data in Greece regarding the community in the mainland as well as studies that focus on insular Greek population^14^. The geographical distribution of Greek islands poses several disadvantages regarding the access of their inhabitants to healthcare services, usually needing to travel to big cities to perform medical examinations. The poor infrastructure allocation in combination with the economic crisis currently in Greece creates a variety of problems in the function of the insular Health Care Centers^15^. The Health Care Centers providing the primary healthcare are usually understaffed and have poor infrastructure. This is the main reason why they have been unable to develop their own local policies and their performance has not yet met sufficiently the medical needs of island inhabitants^16^. This issue was highlighted also during the refugee crisis, nevertheless it is a chronic dysfunction of the Greek Healthcare System^17^.

The aim of the present study was to assess the selfreported community prevalence of OSAS in insular Greece. Given that the access to health services is very limited in the majority of Greek islands, an estimate of the magnitude of the OSAS burden and the level of awareness in the insular population is critical in order to design targeted health promotion strategies for the early detection and treatment of OSAS.

## MATERIAL AND METHODS

### Study Design

Four insular complexes (Ionian Islands, Cyclades, Dodecanese and Northeast Aegean) were studied by means of telephone survey. All inhabited islands belonging to these complexes were included in the study, thus 41 islands in total [7 islands from the Ionian complex (Corfu, Zante, Kefalonia, Lefkada, Paxoi, Kythera, Ithaca), 12 islands from Cyclades (Naxos, Syros, Andros, Paros, Tinos, Mykonos, Milos, Kimolos, Serifos, Sifnos, Santorini, Kea-Kithnos), 15 islands from Dodecanese (Astipalaia, Agathonisi, Kalymnos, Karpathos, Kasos, Kos, Leipsoi, Leros, Megisti, Nisiros, Patmos, Symi, Halki, Tilos, Rhodes) and 7 islands from the Northeastern Aegean islands (Samos, Lesvos, Chios, Ikaria, Limnos, Thasos, Samothraki)]. The overall population of these islands was 738.294 while the population per complex as follows: Ionian Islands 208.972, Cyclades 122.613, Dodecanese 191.272 and Northeast Aegean 215.437. In order to have a representative sample we aimed at including one thousandth of the total population of every island complex. Participant inclusion criteria included adulthood (age >18), permanent residence of the participant in the island and possession of a driver’s license. The research protocol was approved by the Research Committee of the University of Thessaly (protocol number 2800/2017).

### Telephone survey protocol

Seven researchers performed the telephone survey following a standardized protocol. The study was conducted for the period between October 2017 and July 2018. Selection of the subjects was done in a randomized way via internet telephone directory of the Greek Telecommunication Organization (ΟΤΕ–https://www.11888.gr/white-pages/) following a standardized pattern by choosing one telephone number every fifty telephone numbers. Cell phones enlisted were excluded in order to assure that the participants are permanent residents. Initially the subjects were introduced to the nature and the goal of the telephone survey and subsequently were asked to provide their verbal informed consent in order to participate in the survey as anonymous participants. Participants were given the option to withdraw at any point of the telephone survey if they wished to do so. A prerequisite for participation was age above 18 years and possession of a driver’s license. Every questionnaire had to be fully answered in order to be included in the analysis.

### Assessment Tools

The Berlin Questionnaire (BQ) and the Epworth Sleepiness Scale (ESS) were used to evaluate the risk of Obstructive Sleep Apnea (OSA) and Excessive daytime sleepiness (EDS). Both versions used have been translated and adjusted to Greek ^5,6^. BQ provided data regarding snoring incidents, apnea incidents, daily exhaustion and hypertension- BMI consisting of 3 categories accordingly and 10 questions in total. Possible answers for questions of the first 2 categories spanned from 1 (never) to 5 (very often). Score ≥2 in the first two categories was considered as high risk, while in the last category self-reported hypertension and BMI≥30 was interpreted as high risk. Participants with high risk in two out of three categories were considered as being at high risk for suffering from sleep apnea. The ESS questionnaire provided data regarding EDS, consisting of 8 questions which could score 0 to 3 each. Score more than 10 was considered as in high risk for EDS.

To assess the community awareness about OSAS a specific questionnaire was constructed which included 3 questions (yes/no questions) about the participant’s knowledge about OSAS, their knowledge regarding the way of clinical diagnosis and a specific question regarding the knowledge of PSG sleep study that aimed to amplify the reliability of the answer in the second question. Questions about demographics (age, family state, educational level) and smoking habits were also included in this questionnaire.

### Statistical Analysis

Data were tabulated in an Excel spreadsheet and statistical analysis was performed with Graphpad Prism v.7 using the chisquare and t-test where appropriate. Internal consistency of the BQ and ESS was tested with Cronbach’s alpha.

## RESULTS

### Sample Characteristics and Responsiveness Rate

A total of 6538 telephone calls were carried out, out of which 3818 were not responded. Accordingly, 2720 calls were responded by the island’s inhabitants. From this pool of participants 700 were willing to participate in the study and fulfilled the inclusion criteria resulting in a responsiveness rate of 25.74%. The study included 288 males and 412 females (M/F ratio=0.7). The mean age of the participants was 50±14.5 years and the mean BMI was 26.56±4.93. No statistically significant differences were observed among the island complexes regarding gender distribution and age (p>0.05). Detailed characteristics of the participants are shown in Table 1.

**Table 1:** Characteristics of the participants of the study.

	Males (288)	Females (412)	*p* Value
Age (years)	51.86±16.10	48.84±13.20	<0.05
Young adulthood (#)	52	67	
Middle age (#)	114	216	
Older adulthood (#)	122	129	
Height (cm)	175.90±6.56	164.4±5.82	<0.05
Weight (kg)	85.76±13.55	69.61±14.56	<0.05
BMI (kg/m^2^)	27.72±4.26	25.74±5.21	<0.05
Smokers (%)	42.7	20.6	<0.05
Pack years (#)	24.04±23.94	20.79±17.68	0.70

Young adulthood: 18-35; Middle age: 36-55; Older adulthood: 56-above; BMI: Body Mass Index.

### OSAS risk according to BQ and ESS

From a total of 700 participants, 191 scored positive in the BQ (27.29%). Regarding the gender, 102/412 females (24.76%) and 89/288 males (30.90%) were at high risk for OSA. On the other hand, 108/700 participants scored above 10 in the ESS questionnaire (15.43%). Out of these 108 participants, 56 (13.59%) were female and 52 were male (18.06%). From the 700 participants 44 (6.29%) scored positive both in the BQ and ESS questionnaires which qualifies them as being at high risk for OSAS. Out of these 44 participants 19 were females (4.62%) and 25 males (8.68%). Detailed numbers of the high-risk participants per test in each insular complex are given in Table 2. There were no significant differences regarding high risk participants with regards to gender and complex of residence. Cronbach’s alpha was 0.887 and 0.899 for the BQ and ESS respectively in our study sample.

**Table 2:** High Risk Prevalence of participants for OSA, EDS and OSAS.

	BQ High Risk		ESS High Risk		BQ & ESS High Risk	
Gender	M (#)	F (#)	M (#)	F (#)	M (#)	F (#)
Ionian Islands (n=200)	18	23	11	8	5	4
Dodecanese (n=200)	35	26	16	15	12	6
Cyclades (n=116)	17	17	6	16	1	4
Northeast Aegean (n=184)	19	36	19	17	7	5
Total (n=700)	89	102	52	56	25	19
% over total per Gender	30.90%	24.76%	18.06%	13.59%	8.68%	4.62%
% over total	27.29%		15.43%		6.29%	

OSA: Obstructive Sleep Apnea; EDS: Excessive Daytime Sleepiness; OSAS:Obstructive Sleep Apnea Syndrome; BQ: Berlin Questionnaire; ESS: Epworth Sleepiness Scale.

### Participants’ awareness regarding OSAS and its clinical diagnosis

**Figure 1:**
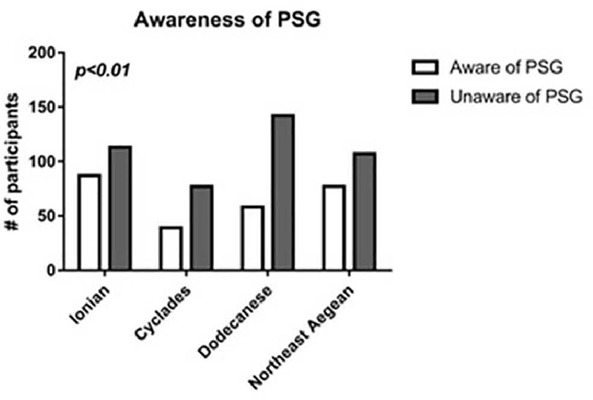
Comparison of the awareness regarding PSG in the 4 insular complexes revealed signiﬁcant differences (p<0.01).

The study participants were asked three questions regarding their knowledge about the existence of OSAS, their knowledge regarding diagnosis and whether they know what a PSG sleep study is or not. From a total of 700 participants, 511 (73%) were aware of the term OSAS, 196 (28%) stated that they knew how the diagnosis of OSAS is established while 261 (37.28) stated that they knew what a PSG sleep study was. In Table 3 the distribution of answers in these 3 questions is presented per insular complex. No significant differences occurred among the insular complexes regarding OSAS awareness (p=0.94) and OSAS diagnosis (p=0.17). However, when it comes to the PSG sleep study, significant differences occurred among the four insular complexes (p=0.01) and more specifically, in the Ionian Islands the participants were more informed about the PSG study as opposed to Dodecanese where the participants were the least informed (Figure 1). In all cases the percentage of men was lower than the percentage of women that were informed regarding OSAS, its diagnosis and PSG.

**Table 3:** Participants Awareness regarding OSAS, its diagnosis and PSG per insular complex.

	Awareness regarding OSAS*		Awareness regarding OSAS Diagnosis$		Awareness regarding PSG[Table-fn TFN5]	
**Gender**	**M (#)**	**F (#)**	**M (#)**	**F (#)**	**M (#)**	**F (#)**
Ionian Islands (n=200)	53	95	19	41	29	58
Dodecanese (n=200)	65	78	21	22	26	32
Cyclades (n=116)	26	60	11	25	11	28
Northeast Aegean (n=184)	51	83	20	37	29	48
Total (n=700)	195	319	71	125	95	166
% over total pergender	67.71%	77.43%	24.65%	30.34%	32.99%	40.30%
% over total	73.43%		28.00%		37.29%	

*Question: Have you ever heard about the Obstructive Sleep Apnea Syndrome?;

^$^Question: Are you aware of the way clinical diagnosis for the Obstructive Sleep Apnea Syndrome is done?;

^@^Question: Do you know what a polysomnographic sleep study is?; OSAS: Obstructive Sleep Apnea Syndrome; PSG: Polysomnography.

## DISCUSSION

The novelty of our study relates to the scarcity of epidemiological data regarding the self-reported community prevalence of OSAS and more specifically insular community prevalence which is a rather understudied portion of the Greek population. To this end, we studied the insular population of 4 island complexes (that included 41 islands) comprising 700 participants in a total population of 738.294 individuals based on the 2011 Greek census. We found, based on self-reported questionnaire, that 27.29% of the population was at high risk for OSA according to BQ. According to the results of ESS, 15.43% of the insular population was found at high risk for EDS. The only published study on community OSA prevalence regarding Mediterranean Basin islands is from Cyprus, an island country. The study assessed the risk of 4118 participants with the STOPBang (SB) questionnaire and reported a prevalence of moderate to high OSA risk of 34% in the general population of Cyprus with no differences in the prevalence between the urban and rural population^18^. Compared to our rural insular sample the prevalence of OSA is lower than Cyprus, however we could consider the result comparable given that the SB questionnaire is more sensitive than the BQ^19^. Other studies that involve community OSA prevalence in the mainland of countries from the Mediterranean Basin originate from Lebanon^20^, and Morocco^21^. In Lebanon in a sample of 501 participants, with similar age (45.2±15.2 years) and female percentage (64%) with our study, 31.3% was found in high-risk for OSA based on BQ results^20^. One difference between the two studies was that our sample was predominantly rural while in the Lebanon study the sample was urban. Still, their result is very close to the one we report in our study in Greek insular population. In a Moroccan study of 503 participants with similar age (42.7±14.1 years) as in our study, the OSAS prevalence was 9.5%^21^. This Moroccan population was not insular but urban and potentially this could explain the significantly higher OSAS prevalence compared to our insular population found at high risk for OSAS that was 6.29%. Still in a recent systematic review a range of 6% to 17% of serious OSAS was estimated, therefore our results agree with the published literature^22^.

Data about the public awareness of these insular regions were also collected. The insular population is considered less likely to be aware of OSAS compared to continental population, as in most cases access to tertiary health care institutions or sleep clinics is far more difficult. It was observed that while 73.43% were aware of OSAS as an entity, a much lower percentage (32.79%) were familiar to PSG sleep study being the golden standard for OSAS diagnosis and even less could name the PSG when asked about the way of OSAS diagnosis (28%). Such variations of awareness among the questions support that it is low amongst the insular population. These differences in the above findings reinforce the notion that focused health promotion programs should be installed aiming at informing and screening the insular population in Greece.

Our study had certain limitations. Being a telephone survey, our study could only make use of the BQ and ESS questionnaires in assessing the risk, and not other available questionnaires such as STOP, SB and the 4 variable screening test (4-V) since they require clinical evaluation of the patient (neck perimeter measurement, blood pressure measurement)^23,24,25^. Pataka et al., described that BQ has lower sensitivity than STOP and SB and lower specificity than 4-V, in a study conducted in a sleep clinic in Greece^19^. Our methodological approach to use only landlines and not cell phones may have introduced some bias in our study towards older ages of participants. Furthermore, no PSG study evaluation was provided in this study, therefore more in-depth study is required to have a definite answer regarding the prevalence of OSAS in the insular population of Greece.

## CONCLUSION

We reported novel findings regarding the self-reported community prevalence of OSA, EDS and OSAS in adult insular Greek population that are in accordance with similar published studies in Cyprus, an island country, as well as Morocco and Lebanon that are Mediterranean country in proximity to Greek islands. This is the first study to provide such information on insular population in Greece comprising 41 Greek islands. Furthermore, we were able to demonstrate the need of installation of OSAS related health promotion programs in the Greek islands since the level of awareness was low especially regarding OSAS diagnosis.
